# Concrete Containing Waste Glass as an Environmentally Friendly Aggregate: A Review on Fresh and Mechanical Characteristics

**DOI:** 10.3390/ma15186222

**Published:** 2022-09-07

**Authors:** Shaker Qaidi, Hadee Mohammed Najm, Suhad M. Abed, Yasin Onuralp Özkılıç, Husam Al Dughaishi, Moad Alosta, Mohanad Muayad Sabri Sabri, Fadi Alkhatib, Abdalrhman Milad

**Affiliations:** 1 Department of Civil Engineering, College of Engineering, University of Duhok, Duhok 42001, Iraq; 2 Department of Civil Engineering, Zakir Husain Engineering College, Aligarh Muslim University, Aligarh 202002, India; 3 Department of Highways & Airports Engineering, College of Engineering, University of Diyala, Baqubah 32001, Iraq; 4 Department of Civil Engineering, Faculty of Engineering, Necmettin Erbakan University, Konya 42000, Turkey; 5 Department of Civil and Environmental Engineering, College of Engineering, University of Nizwa, P.O. Box 33, Nizwa 616, Oman; 6 Peter the Great St. Petersburg Polytechnic University, 195251 St. Petersburg, Russia; 7 Department of Structural Engineering, Faculty of Civil Engineering and Built Environment, Universiti Tun Hussein Onn Malaysia (UTHM), Parit Raja 86400, Malaysia

**Keywords:** waste glass, recycling, construction materials, sustainable concrete, mechanical properties

## Abstract

The safe disposal of an enormous amount of waste glass (WG) in several countries has become a severe environmental issue. In contrast, concrete production consumes a large amount of natural resources and contributes to environmental greenhouse gas emissions. It is widely known that many kinds of waste may be utilized rather than raw materials in the field of construction materials. However, for the wide use of waste in building construction, it is necessary to ensure that the characteristics of the resulting building materials are appropriate. Recycled glass waste is one of the most attractive waste materials that can be used to create sustainable concrete compounds. Therefore, researchers focus on the production of concrete and cement mortar by utilizing waste glass as an aggregate or as a pozzolanic material. In this article, the literature discussing the use of recycled glass waste in concrete as a partial or complete replacement for aggregates has been reviewed by focusing on the effect of recycled glass waste on the fresh and mechanical properties of concrete.

## 1. Introduction

**Figure 1:**
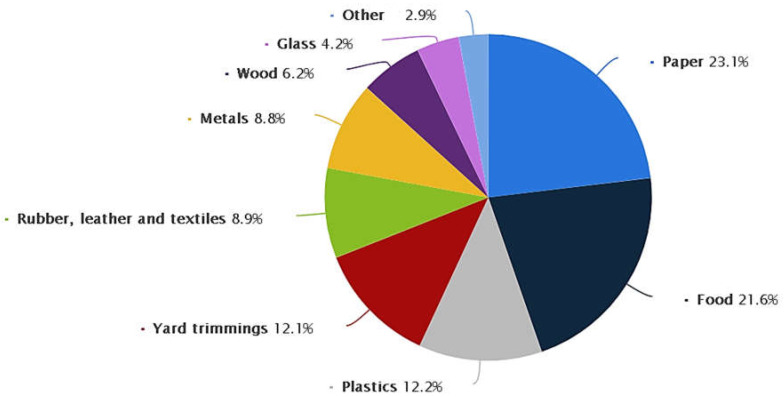
Distribution of MSW stream produced in the U.S in 2018. Adapted from [7].

Glass is one of the world’s most diverse substances because of its substantial properties, such as chemical inertness, optical clarity, low permeability, and high authentic strength [1,2,3]. The usage of glass items has greatly increased, leading to enormous quantities of WG. Globally, it is estimated that 209 million tons of glass are produced annually [4,5,6]. In the U.S., according to the Environmental Protection Agency (EPA) [7,8,9], 12.27 million tons of glass were created in 2018 in municipal solid waste (MSW), as shown in Figure 1, most of which were containers for drinking and food. Furthermore, in 2018, the EU generated 14.5 million tons of glass package wastes [10,11,12]. The quantity of generated WG will increase due to the increasing demand for glass components [13,14,15,16].

Recycling and reducing waste are key parts of a waste-management system since they contribute to conserving natural resources, reducing requests for waste landfill space, and reducing pollution of water and air [17,18]. According to Meyer [19], by 2030, the EU zero-waste initiative estimates that improvements in resource efficiency throughout the chain could decrease material input requirements by 17% to 24%, satisfying the demand for raw materials between 10% to 40%, and could contribute to reducing emissions by 40% [20,21,22].

In fact, innovative options for recycling WG must be developed. One significant option is to use WG for construction materials [23]. The recycling of WG not only decreases the demand for landfill sites in the building sector but also significantly helps in decreasing the carbon footprint and saving resources [24,25,26]. In 1963, Schmidt and Saia [27] performed the first research on the use of WG for building materials. The authors recycled WG into useful glass particles for wall-panel production. Subsequently, a significant study was conducted in order to use recycled glass for fine or coarse aggregate in mortar and concrete, because of the good hardness of the glass [14,28,29]. This study aims at reviewing the possibilities of utilizing WG in concrete as a partial or full replacement for fine or coarse aggregates in order to give practical and brief guidance on recycling and using WG [30,31,32,33].

## 2. Research Significance

Besides the above-mentioned dangers of WG and the need to recycle it economically and environmentally, this research explores the source of WG as well as its physical and chemical characteristics. In addition, this study aims to review the literature that discusses the use of recycled WG in concrete as a partial or complete alternative to aggregates by focusing on the effect of this waste on the fresh and mechanical properties of concrete in order to demonstrate the possibilities of using recycled WG in concrete and to provide practical and brief guidance. Furthermore, we are establishing a foundation for future study on this material and describing research insights, existing gaps, and future research goals.

## 3. Properties of Glass

### 3.1. Chemical Properties of Glass

Glass exists in various colors and types, with various chemical components. Table 1 and Table 2 show the chemical compositions of different colors and types of typical glass, respectively.

### 3.2. Physical and Mechanical Properties of Glass

The physical and mechanical properties of crushed WG are listed in Table 3 and Table 4, respectively.

## 4. Fresh Concrete Properties

### 4.1. Workability

There are two parallel points of view on the workability of WG-containing concrete. A review of past studies on the impact of WG aggregates on the mixes of workability is summarized in Table 5. It can be noticed that various research investigations have shown that the mixing of WG increases workability. They connected this beneficial impact of WG on the workability to the weaker cohesive between the cement mortar and the smooth surfaces of waste glass [48,49,50,51,52]. The smooth surface and low absorption capacity of WG are also important factors in increasing workability [53,54]. For example, Ali and Al-Tersawy [55] substitute fine aggregate in self-compacting concrete (SCC) mixes with recycled WG at levels of 10% to 50% by volume. Constant content of water–cement ratio and various superplasticizer doses have been used. They stated that slump flow increased by 2%, 5%, 8%, 11%, and 85%, with the incorporating of 10%, 20%, 30%, 40% and 50% of WG, respectively. In addition, Liu, Wei, Zou, Zhou and Jian [56] substitute fine aggregate in ultra-high-performance concrete (UHPC) mixes with recycled liquid crystal display (CRT) glass at levels of 25% to 100% by volume. Constant content of water–cement ratio and various superplasticizer (SP) doses have been used. Moreover, they stated that flowability increased by 11, 14, 16, and 12 mm, compared to the control sample, incorporating 25%, 50%, 75%, and 100% WG, respectively. Enhancing the workability by including WG is a benefit of utilizing this recycled material [57,58,59,60]. There is potential to utilize glass to create HPC in which high workability is necessary. In addition, WG can be used to boost workability rather than employing admixtures such as HRWR or superplasticizers [61,62,63,64].

Contrastingly, some studies have stated that including waste glass into the mixes lowered workability. Nevertheless, such a decrease has been associated with sharp edges, higher glass particle aspect ratio, and angular form, with obstruction of the movement of particles and cement mortar [65,66,67,68,69,70,71]. For example, Wang [72] substitutes fine aggregate in liquid crystal display glass concrete (LCDGC) mixes with recycled LCD at levels of 20% to 80% by volume. Various contents of w/c ratio (0.38–0.55) and various superplasticizer doses have been used. The author stated that slump flow decreased by 4%, 7%, 19%, and 26%, incorporating 20%, 40%, 60%, and 80% of WG, respectively, for w/c of 0.44. In addition, Arabi, Meftah, Amara, Kebaïli and Berredjem [73] substitute coarse aggregate in SCC mixes with recycled windshield glass at levels of 25% to 10% by volume. Various contents of w/c ratio (0.60–0.69) and various superplasticizer doses have been used. They stated that slump flow decreased by 3%, 8%, 9%, and 11%, incorporating 20%, 40%, 60%, and 80% of WG, respectively. According to Rashad [61], the optimal content of glass waste to achieve good workability is 20%.

**Table 5:** Summary of the results of past studies on the workability of waste-glass concrete.

Refs.	Type of Composite	Source	Type of Sub.	WG Sub. Ratio%	WG Size (mm)	w/c or w/b	Addit. or Admix.	Outcomes
[74]	SCGC	LCD	F.A	10, 20, & 30 (vol.%)	11.8	0.28	SP	Slump flow increased by 11%, 17%, and 21%, respectively.
[75]	HPGC	LCD	F.A	10, 20, & 30 (vol.%)	0.149–4.75	0.25, 0.32, & 0.34	SP	Slump flow increased, ranged between 7–9%.
[76]	Steel slag concrete	WG	C.A	16.5 & 17.5 (vol.%)	4.9–10 & 4.9–16	0.4 & 0.55	WR	Slump increased by 167%, for substitution 16.5% (w/c of 0.55, and size of 4.9–10 mm). Slump increased by 8%, for substitution 17.5% (w/c of 0.40, and size of 4.9–16 mm).
[77]	Cement concrete	WG & PVC	F.A	5, 10, 15, 20, 25, & 30 (wt.%)	0.15–0.6	0.44, 0.5, & 0.55	-	Slump value changed by −7%, +33%, +47%, +31, +36, and +40%, respectively, for w/c of 0.5.
[78]	Waste glass concrete	WG	F.A	18, 19, 20, 21, 22, 23, & 24 (vol.%)	0.15–0.6	0.4	SP	Workability decreased by increasing the WG ratio.
[79]	Waste glass concrete	CRT	F.A	50 & 100 (vol.%)	≤5	0.35, 0.45, & 0.55	WR & AE	Slump increased by 55%, and 115%, respectively, for w/c of 0.45.
[80]	Waste glass concrete	WG	C.A	10, 20, & 30 (wt.%)	≤20	0.55	-	Slump decreased by 3%, 5%, and 9%, respectively.
[73]	SCC	Windshield	C.A	25, 50, 75, & 100 (vol.%)	9.5 & 12.7 (mixed)	0.6–0.69	Marble filler & SP	Slump flow decreased by 3%, 8%, 9%, and 11%, respectively.
[81]	UHPC	WG	F.A	25, 50, 75, & 100 (wt.%)	≤0.6	0.19	Steel fiber & HRWRA	Slump increased by 25%, 111%, 321%, and 532%, respectively.
[56]	UHPC	CRT	F.A	25, 50, 75, & 100 (vol.%)	0.6–1.18	0.19	Steel fiber, SF, & SP	Flowability increased by 11, 14, 16, and 12 mm, respectively, compared to control (200 mm).
[82]	Waste glass concrete	WG	F.A	15 & 30 (vol.%)	≤4.75	0.5	-	Slump decreased by 9%, and 39%, respectively.
[83]	Waste-based concrete	WG	F.A	100 (vol.%)	≤1.9	0.47	SP & GBFS	Glass sand showed lower workability compared to Lead smelter slag (LSS).
[84]	Waste glass concrete	WG	F.A	5, 15, & 20 (vol.%)	0.15–4.75	0.55	-	Slump decreased by 19%, 29%, and 35%, respectively.
[55]	SCC	WG	F.A	10, 20, 30, 40, & 50 (vol.%)	0.075–5	0.4	SF & SP	Slump flow increased by 2%, 5%, 8%, 11%, and 85%, respectively.
[85]	Cement concrete	WG	F.A	5, 10, 15, & 20 (vol.%)	0.15–9.5	0.56	-	Slump decreased by 1%, 3%, 4%, and 5%, respectively.
[86]	Waste glass concrete	Waste E-glass	F.A	10, 20, 30, 40, & 50 (wt.%)	≤4.75	0.68	SF & F.A.	Slump decreased by 2%, 1%, 50%, 55, and 54%, respectively.
[87]	Waste glass concrete	WG	F.A	10, 15, & 20 (vol.%)	0.15–4.75	0.52	-	Slump decreased by 24%, 23%, and 33%, respectively.
[88]	Waste glass concrete	WG	F.A	15, 20, 30, & 50 (wt.%)	≤5	0.52, 0.57, & 0.67	-	Slump decreased by 0%, 0%, 13%, and 13%, respectively, for w/c of 0.57.
[89]	Waste glass concrete	Green waste glass	F.A	30, 50, & 70 (wt.%)	≤5	0.5	AE	Workability decreased, ranged between 19–44%.
[65]	Waste glass concrete	Soda-lime glass	F.A	50 & 100 (vol.%)	≤5	0.38	MK	Slump decreased by 0%, and 38%, respectively.
[48]	Waste glass concrete	WG	F.A & C.A	10, 25, 50, & 100 (vol.%)	N.M	0.48	-	Slump value changed by −6%, +6%, +18%, and +6%, respectively.
[72]	LCDGC	LCD	F.A	20, 40, 60, & 80 (vol.%)	≤4.75	0.38, 0.44, & 0.55	-	Slump flow decreased by 4%, 7%, 19%, and 26%, respectively.
[90]	Cement concrete	LCD	F.A	20, 40, 60, & 80 (vol.%)	≤4.75	0.48	-	Slump value changed by 0%, −5%, −5%, and +20%, respectively.
[91]	Alkali-activated mortar	Cullet	F.A	25, 50, 75, & 100 (vol.%)	≤2.36	0.6	F.A., GBFS, SH, & SS	Flowability increased, ranged between 4–15%.
[92]	Waste glass concrete	WG	F.A	25, 50, 75, & 100 (wt.%)	≤5	0.5	-	Slump decreased by 9%, 7%, 15%, and 27%, respectively.

Where: SCGC is self-compacting glass concrete; SCC is self-compacting concrete; HPGC is high performance recycled liquid crystal glasses concrete; UHPC is ultra-high performance concrete; LCDGC is liquid crystal display glass concrete; LCD is liquid crystal display; CRT is cathode ray tube; WG is waste glass; PVC is polyvinyl chloride; SP is superplasticizer; HRWRA is a high-range water-reducing agent; WR is water-reducing; AE is air-entraining; SF is silica fume; F.A. is fly ash; GBFS is granulated blast furnace slag; MK is metakaolin; SH is sodium hydroxide solution; SS is sodium silicate solution; F.A is fine aggregate; C.A is coarse aggregate; vol. is replacing by volume; wt. is replacing by weight.

### 4.2. Bulk Density

**Figure 2:**
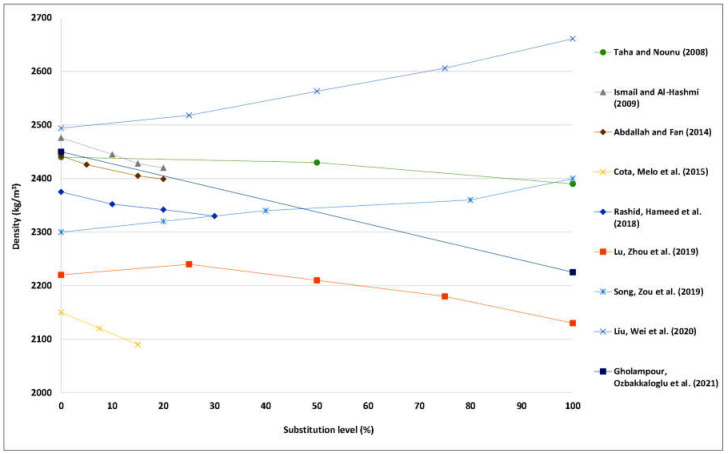
Bulk density of concrete with various content of WG. Adapted from references [56,65,80,83,84,87,105,106,107].

Past studies on the impact of WG aggregates on the bulk density, which are summarized in Figure 2, revealed that the majority of studies showed that incorporating glass waste into mixtures reduces density. This decrease can be ascribed to the lesser density of WG compared to natural aggregate [42,65,93,94], as well as the lower specific gravity [43,66,87,93,95]. For example, Taha and Nounu [65] substitute fine aggregate in waste-glass concrete (WGC) mixes with recycled soda-lime glass at levels of 50% to 100% by volume. They stated that the fresh density of WG concrete mixes reduced by 1% and 2% incorporating 50% and 100% of WG, respectively. This density drop might be realized as one benefit of using this material in concrete for engineering purposes [96,97,98,99].

On the other hand, Liu, Wei, Zou, Zhou and Jian [56] stated that concrete of 10 to 50% WG had a fresh density greater than reference. The authors substitute F.A in UHPC mixes with recycling CRT glass at levels of 25% to 100% by volume. They stated that the fresh density of waste-glass concrete mixtures increased by 1% 2.5%, 3.5%, and 6%, incorporating 25%, 50%, 75%, and 100% of WG, respectively. The authors attributed the reason to the fact that the density of CRT glass (2916 kg/m^3^) was larger than that of fine aggregate (2574 kg/m^3^) [100,101,102,103,104].

## 5. Mechanical Properties

### 5.1. Compressive Strength

By reviewing past studies on the impact of WG aggregates on the compressive strength of waste-glass concrete, summarized in Table 6, it can be noticed that most studies shown that incorporating glass waste into concrete reduces compressive strength. The researchers ascribed this behavior to (i) the sharp edges and smooth particle surfaces, leading to a poorer bond between cement mortar and glass particles at the interfacial transition zone (ITZ) [14,40,42,43,55,66,87,90,108,109]; (ii) increased water content of the glass aggregate mixes due to the weak ability of WG to absorb water [43,110]; and (iii) the cracks caused by expanding stress formed by the alkali-silica reaction produced from the silica in WG [40]. For example, Park, Lee and Kim [89] substitute fine aggregate in WGC with recycled green WG at levels of 30% to 70% by weight. They stated that the compressive strength of concrete decreased by 3%, 13%, and 18%, incorporating 30%, 50%, and 70% of WG, respectively. In addition, Terro [48] noted that concrete, which contains up to 25% of WG, showed compressive strength values greater than the reference, whereas concrete with a substitution level of over 25% declined in compressive strength.

In order to better understand the impact of glass waste on the properties of the waste-glass concrete [111,112,113,114]. Omoding, Cunningham and Lane-Serff [115] investigated the concrete microstructure via SEM by replacing between 12.5–100% of the coarse aggregate with green waste glass with a size of 10–20 mm. The authors stated (i) that there is a weak connection between the waste glass and the cement matrix. This is because of a reduction in bonding strength between the waste glass and the cement paste because of the high smoothness of waste glass, consequently resulting in cracks and poor adherence between waste glass and cement paste; and (ii) as the content of waste glass increases, the proportion of cracks and voids increases in the concrete’s matrix.

However, some studies have stated that waste glass increases mechanical strength. This increase is primarily realized because of the surface texture and strength of the waste glass particles compared to natural sand [116,117,118] and the pozzolanic reaction of waste glass aggregate [119,120,121]. For example, Jiao, Zhang, Guo, Zhang, Ning and Liu [81] substitute fine aggregate in UHPC with recovered WG at levels of 25% to 100% by weight. They stated that the compressive strength of concrete increased by 2%, 17%, 34%, and 20%, incorporating 25%, 50%, 75%, and 100% WG, respectively.

Regarding the influence of WG color on properties, some studies have stated that the color of WG did not produce any noticeable variation in strength [89,122]. On the contrary, Tan and Du [66] claimed that clear waste glass showed less strength.

**Table 6:** Summary of the results of past studies on the compressive strength of waste-glass concrete.

Refs.	Type of Composite	Source	Type of Subs.	WG Subs. Ratio	WG Size (mm)	w/c or w/b	Addit. or Admix.	Com. Str. of Control (MPa)	Outcomes
[74]	SCGC	LCD	F.A	10, 20, & 30 (vol.%)	11.8	0.28	SP	65	Decreased by 2%, 5%, and 3%, respectively.
[75]	HPGC	LCD	F.A	10, 20, & 30 (vol.%)	0.149–4.75	0.25, 0.32, & 0.34	SP	56	Decreased by 25%, 32%, and 29%, respectively, for w/c of 0.32.
[123]	Autoclaved aerated concrete	CRT	F.A	5 & 10 (vol.%)	2.16–3.3	N.M	-	29	Decreased by 2%, and 0%, respectively.
[77]	Cement concrete	WG & PVC	F.A	5, 10, 15, 20, 25, & 30 (wt.%)	0.15–0.6	0.44, 0.5, & 0.55	-	34	Decreased by 1%, 4%, 4%, 6%, 7%, and 9%, respectively, for w/c of 0.50.
[78]	Waste glass concrete	WG	F.A	18, 19, 20, 21, 22, 23, & 24 (vol.%)	0.15–0.6	0.4	SP	33	Changed by +6%, +9%, +12%, +9%, +3%, −6% and −9%, respectively.
[79]	Waste glass concrete	CRT	F.A	50 & 100 (vol.%)	≤5	0.35, 0.45, & 0.55	WR & AE	28	Decreased by 21%, and 32%, respectively, for w/c of 0.45.
[80]	Waste glass concrete	WG	C.A	10, 20, & 30 (wt.%)	≤20	0.55	-	24	Decreased by 13%, 15%, and 23%, respectively.
[105]	Waste glass concrete	WG	F.A	25, 75, & 100 (wt.%)	0.15–5	0.48–0.66	-	38	Changed by +5%, +8%, +3%, and −8%, respectively.
[124]	Waste glass concrete	Cullet	C.A	25, 50, & 75 (wt.%)	2.36–5	0.29	SF	32	Decreased by 6%, 3%, 22%, and 25%, respectively.
[73]	SCC	Windshield	C.A	25, 50, 75, & 100 (vol.%)	9.5 & 12.7	0.6–0.69	Marble filler & SP	33	Decreased by 15%, 24%, 24%, and 30%, respectively.
[125]	HSPC	WG	C.A	25, 50, 75, & 100 (vol.%)	2.36–5	0.14	SF & SP	50	Decreased by 4%, 20%, 30%, and 36%, respectively.
[81]	UHPC	WG	F.A	25, 50, 75, & 100 (wt.%)	≤0.6	0.19	Steel fiber & HRWRA	108	Increased by 2%, 17%, 34%, and 20%, respectively.
[56]	UHPC	CRT	F.A	25, 50, 75, & 100 (vol.%)	0.6–1.18	0.19	Steel fiber, SF, & SP	180	Decreased by 7%, 11%, 16%, and 18%, respectively.
[115]	Glass aggregate concretes	WG	C.A	12.5, 25, 50, & 100 (vol.%)	10–20	0.52	SP	45	Decreased by 4%, 16%, 20%, and 27%, respectively.
[82]	Waste glass concrete	WG	F.A	15 & 30 (vol.%)	≤4.75	0.5	-	48	Decreased by 6%, and 0%, respectively.
[84]	Waste glass concrete	WG	F.A	5, 15, & 20 (vol.%)	0.15–4.75	0.55	-	33	Decreased by 6%, 3%, and 0%, respectively.
[55]	SCC	WG	F.A	10, 20, 30, 40, & 50 (vol.%)	0.075–5	0.4	SF & SP	62	Decreased by 5%, 15%, 18%, 23%, and 24%, respectively.
[85]	Cement concrete	WG	F.A	5, 10, 15, & 20 (vol.%)	0.15–9.5	0.56	-	32	Increased by 9%, 44%, 25%, and 38%, respectively.
[87]	Waste glass concrete	WG	F.A	10, 15, & 20 (vol.%)	0.15–4.75	0.52	-	44	Changed by −9%, −9%, and +5%, respectively.
[88]	Waste glass concrete	WG	F.A	15, 20, 30, & 50 (wt.%)	≤5	0.52, 0.57, & 0.67	-	48	Decreased by 2%, 4%, 13%, and 19%, respectively, for w/c of 0.57.
[89]	Waste glass concrete	Green waste glass	F.A	30, 50, & 70 (wt.%)	≤5	0.5	AE	38	Decreased by 3%, 13%, and 18%, respectively.
[48]	Waste glass concrete	WG	F.A & C.A	10, 25, 50, & 100 (vol.%)	N.M	0.48	-	40	Changed by +38%, +3%, −5%, and −50%, respectively.
[72]	LCDGC	LCD	F.A	20, 40, 60, & 80 (vol.%)	≤4.75	0.38, 0.44, & 0.55	-	39	Decreased by 3%, 10%, 13%, and 15%, respectively, for w/c of 0.44.
[90]	Cement concrete	LCD	F.A	20, 40, 60, & 80 (vol.%)	≤4.75	0.48	-	36	Decreased by 6%, 11%, 22%, and 25%, respectively.
[107]	Waste glass concrete	CRT	F.A	20, 40, 60, 80, & 100 (vol.%)	4.75	0.45	F.A.	38	Decreased by 5%, 8%, 8%, 11%, and 13%, respectively.
[126]	Resin concretes	WG	F.A	0–100 (wt.%)	≤2	N.M	Epoxy resin	95	Decreased by 33%, for substitution of 100%.
[127]	Concrete blocks	WG	F.A	100 (vol.%)	4.75, 2.36, 1.18, & 0.6	0.23	-	34	Decreased by 18%.
[91]	Alkali-activated mortar	Cullet	F.A	25, 50, 75, & 100 (vol.%)	≤2.36	0.6	F.A., GBFS, SH, & SS	70	Decreased by 3%, 6%, 7%, and 10%, respectively.
[128]	Waste glass concrete	WG	F.A	25, 50., 75, & 100 (wt.%)	≤5	0.5	-	20	Changed by +20%, +15%, −10%, and −35%, respectively.

Where: SCGC is self-compacting glass concrete; SCC is self-compacting concrete; HPGC is high performance recycled liquid crystal glasses concrete; HSPC is high-strength pervious concrete; UHPC is ultra-high performance concrete; LCDGC is liquid crystal display glass concrete; LCD is liquid crystal display; CRT is cathode ray tube; WG is waste glass; PVC is polyvinyl chloride; SP is superplasticizer; HRWRA is a high-range water-reducing agent; WR is water-reducing; AE is air-entraining; SF is silica fume; F.A. is fly ash; GBFS is granulated blast furnace slag; MK is metakaolin; SH is sodium hydroxide solution; SS is sodium silicate solution; F.A is fine aggregate; C.A is coarse aggregate; vol. is replacing by volume; wt. is replacing by weight.

### 5.2. Splitting Tensile Strength

Past studies on the impact of WG aggregates on the splitting tensile strength of waste-glass concrete, which are summarized in Table 7, revealed that incorporating glass waste into concrete reduces tensile strength. Similarly, as in compressive strength, studies have attributed the main reason for this behavior to the poor bond between cement paste and glass particles at the ITZ. For example, Wang [72] substitutes fine aggregate in liquid crystal display glass concrete (LCDGC) with recycled LCD glass at levels of 20% to 80% by volume. The author stated that splitting tensile strength of concrete decreased by 1%, 7%, 8%, and 9%, incorporating 20%, 40%, 60%, and 80% of WG, respectively, for w/c of 0.44. Moreover, Ali and Al-Tersawy [55] substitute fine aggregate in self-compacting concrete (SCC) with recycled WG at levels of 10% to 50% by volume. They stated that tensile strength of waste-glass concrete decreased by 9%, 15%, 16%, 24%, and 28% incorporating 10%, 20%, 30%, 40%, and 50% of WG, respectively [129,130,131,132].

In contrast, Jiao, Zhang, Guo, Zhang, Ning and Liu [81] indicated that concrete of 25% to 100% WG had a tensile strength greater than reference. The authors substitute fine aggregate in ultra-high-performance concrete (UHPC) with recycled WG at levels of 25% to 100% by weight. They stated that the splitting tensile strength of concrete increased by 1%, 3%, 11%, and 7%, incorporating 25%, 50%, 75%, and 100% of WG, respectively. The author attributed the reason to the effect of using steel fibers.

**Table 7:** Summary of the results of past studies on the splitting tensile strength of waste-glass concrete.

Refs.	Type of Composite	Source	Type of Sub.	WG Sub. Ratio%	WG Size (mm)	w/c or w/b	Addit. or Admix.	Split ten. str. of Control (MPa)	Outcomes
[81]	UHPC	WG	F.A	25, 50, 75, & 100 (wt.%)	≤0.6	0.19	Steel fiber & HRWRA	11.7	Increased by 1%, 3%, 11%, and 7%, respectively.
[82]	Waste glass concrete	WG	F.A	15 & 30 (vol.%)	≤4.75	0.5	-	4.5	Changed by +4%, and −1%, respectively.
[84]	Waste glass concrete	WG	F.A	5, 15, & 20 (vol.%)	0.15–4.75	0.55	-	2.5	Increased by 4%, 12%, and 24%, respectively.
[55]	SCC	WG	F.A	10, 20, 30, 40, & 50 (vol.%)	0.075–5	0.4	SF & SP	6.8	Decreased by 9%, 15%, 16%, 24%, and 28%, respectively.
[85]	Cement concrete	WG	F.A	5, 10, 15, & 20 (vol.%)	0.15–9.5	0.56	-	3.9	Decreased by 0%, 8%, 15%, and 23%, respectively.
[133]	Waste glass concrete	WG	F.A	10, 20, 30, & 40 (wt.%)	≤4.75	0.45	-	2.5	Decreased by 2%, 8%, 10%, and 12%, respectively.
[72]	LCDGC	LCD	F.A	20, 40, 60, & 80 (vol.%)	≤4.75	0.38, 0.44, & 0.55	-	2.38	Decreased by 1%, 7%, 8%, and 9%, respectively, for w/c of 0.44.
[107]	Waste glass concrete	CRT	F.A	20, 40, 60, 80, & 100 (vol.%)	4.75	0.45	F.A.	4.48	Decreased by 6%, 6%, 13%, 15%, and 19%, respectively.
[128]	Waste glass concrete	WG	F.A	25, 50., 75, & 100 (wt.%)	≤5	0.5	-	3.6	Decreased by 22%, 39%, 39%, and 44%, respectively.

Where: UHPC is ultra-high-performance concrete; LCDGC is liquid crystal display glass concrete; LCD is liquid crystal display; CRT is cathode ray tube; WG is waste glass; SP is superplasticizer; HRWRA is a high-range water-reducing agent; SF is silica fume; F.A. is fly ash; F.A is fine aggregate; C.A is coarse aggregate; vol. is replacing by volume; wt. is replacing by weight.

### 5.3. Flexural Strength

The flexural strength of waste-glass concrete shows comparable tendencies to its compressive strength and tensile strength. Most of the research revealed that introducing WG aggregates reduced flexural strength. However, other research showed that flexural strength increased when WG was included [134,135,136]. For instance, Kim, Choi and Yang [79] substitute fine aggregate in WGC with recycled CRT glass at levels of 50% to 100% by volume. They stated that flexural strength of concrete decreased by 9% and 14%, incorporating 50% and 100% of WG, respectively, for w/c of 0.45. On the contrary, Jiao, Zhang, Guo, Zhang, Ning and Liu [81] substitute fine aggregate in UHPC with recovered WG at levels of 25% to 100% by weight. They stated that flexural strength of concrete increased by 2%, 1%, 5%, and 1%, incorporating 25%, 50%, 75%, and 100% of WG, respectively.

Moreover, it can be concluded that the discrepancy between studies may be related to the type, size, and source of WG used in the mixtures. The mineral composition varies as the type of glass changes. Therefore, changing the mechanisms of interaction with binders in concrete, in turn, affects the properties. Table 8 presents the outcomes of various studies on the flexural strength of waste-glass concrete.

### 5.4. Modulus of Elasticity (MOE)

The modulus of elasticity of concrete (MOE) depends on the normal and lightweight aggregates elasticity modulus, cement matrix, and their relative ratios in the mixes [39]. In general, the incorporation of WG aggregates into concrete increases the modulus of elasticity [72,84]. For instance, Steyn, Babafemi, Fataar and Combrinck [82] substitute fine aggregate in WGC with recovered WG at levels of 15% to 30% by volume. They stated that MOE of concrete increased by 1%, and 7%, incorporating 15% and 30% of WG, respectively. In addition, Omoding, Cunningham and Lane-Serff [115] substitute coarse aggregate in glass aggregate concretes with recycled WG at levels of 12.5% to 100% by volume. They stated that MOE of concrete increased by 2% to 4% for a replacement rate of 12.5% to 50%, then decreased by 3% to 9% for replacement ratios above 50% [137,138].

**Figure 3:**
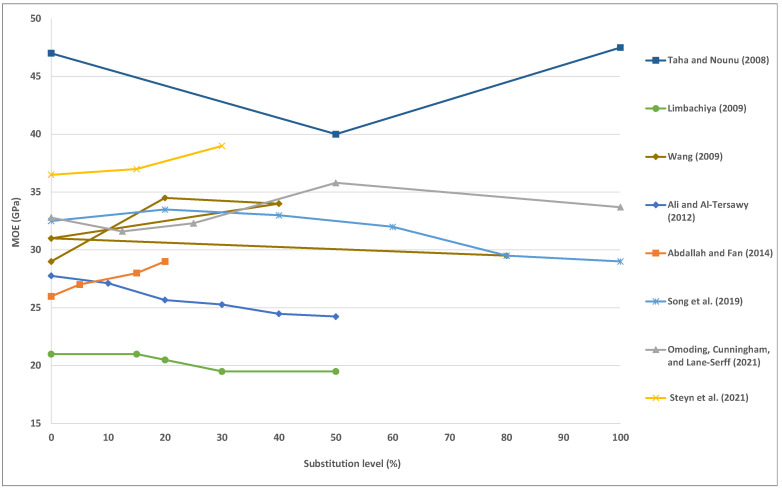
Modulus of elasticity of concrete with various contents of the waste glass. Adapted from references [55,65,72,82,84,88,107,115].

However, some studies have stated that including WG decreases the MOE of concrete. For instance, Ali and Al-Tersawy [55] substitute fine aggregate in SCC with recovered WG at levels of 10% to 50% by volume. They stated that MOE of concrete decreases by 2%, 8%, 9%, 12%, and 13%, incorporating 10%, 20%, 30%, 40% and 50% of WG, respectively. Figure 3 presents the outcomes of various studies on the MOE of WG concrete.

## 6. Conclusions

The utilization of WG in concrete affects the fresh and mechanical properties of waste-glass concrete, which must be taken into consideration before being used in structures. The overall conclusions of this review are:

1. The workability of waste-glass-containing concrete mixtures for fine or coarse aggregates was less than for natural aggregate-containing mixtures. Nevertheless, despite the poorer workability, some studies found that the mixtures were still workable.
2. Most studies indicated that with the introduction of WG, the density of concrete decreased due to the decreased density and specific gravity of waste glass aggregates.
3. The findings of the literature have been somewhat indecisive regarding the properties of concrete, such as compressive strength, splitting tensile strength, flexural strength, and modulus of elasticity.
4. The findings revealed that the compressive strength, splitting tensile strength, and flexural strength of concrete deteriorated by integrating WG. Nevertheless, the findings concerning the elastic modulus of concrete were conflicting. This decrease was essential because of the sharp edges and smooth surface of the waste glass that caused the poorer bond between cement mortar and waste glass particles at the ITZ.
5. Studies also showed that the optimal aggregate substitution level was about 20%. In addition, the glass color does not have a substantial influence on the strength. Although the results are indecisive, WG has the possibility to be an acceptable substitute for fine or coarse concrete aggregates in concrete.
6. Adding waste glass to the concrete mixture may improve certain mechanical characteristics of concrete, reduce concrete dead load, and provide an ecological substitute for normal aggregates.

## 7. Recommendations

This paper makes the following broad recommendations for future investigations:

1. More investigation is required into the mechanical characteristics of high-performance and high-strength waste-glass concrete.
2. The effects of different glass kinds and colors on concrete mixes should be thoroughly investigated in the future.
3. Test fewer common types of glass as aggregates in concrete because the vast majority of research only covers soda-lime glass.
4. Conduct a comprehensive evaluation of the real environmental effects through life-cycle assessment to evaluate the feasibility of using this waste.

## Figures and Tables

**Table 1:** Chemical components of glass for various colors.

Color	Chemical Compositions											Refs.
	SiO_2_	CaO	Na_2_O	Al_2_O_3_	MgO	Fe_2_O_3_	K_2_O	SO_3_	TiO_2_	Cr_2_O_3_	Others	
White	70.39	6.43	16.66	2.41	2.59	0.32	0.23	0.19	0.08	-	0.04 (MnO), 0.02 (Cl)	[34]
Clear	72.42	11.50	13.64	1.44	0.32	0.07	0.35	0.21	0.035	0.002	-	[35]
Flint	70.65	10.70	13.25	1.75	2.45	0.45	0.55	0.45	-	-	-	[36]
Amber	70.01	10.00	15.35	3.20	1.46	-	0.82	0.06	0.11	-	0.04 (MnO)	[34]
Brown	71.19	10.38	13.16	2.38	1.70	0.29	0.70	0.04	0.15	-	-	[37]
Green	72.05	10.26	14.31	2.81	0.90	-	0.52	0.07	0.11	-	0.04 (MnO)	[34]

**Table 2:** Chemical components of glass for various types. Adapted from [38,39].

Type	Uses	Chemical Compositions									
		SiO_2_	K_2_O	Na_2_O	Al_2_O_3_	MgO	PbO	BaO	CaO	B_2_O_3_	Others
Barium glasses	Optical-dense barium crown	36			4		41			10	9% ZnO
	Color TV panel	65	9	7	2	2	2	2	2		10% SrO
Soda-Lime Glasses	Containers	66–75	0.1–3	12–16	0.7–7	0.1–5			6–12		
	Light bulbs	71–73									
	Float sheet	73–74									
	Tempered ovenware	0.5–1.5								13.5–15	
Lead glasses	Color TV funnel	54	9	4	2		23				
	Electronic parts	56	9	4	2		29				
	Neon tubing	63	6	8	1		22				
	Optical dense flint	32	2	1			65				
Aluminosilicate glasses	Combustion tubes	62		1	17	7			8	5	
	Resistor substrates	57			16	7		6	10	4	
	Fiberglass	64.5		0.5	24.5	10.5					
Borosilicate	Chemical apparatus	81		4	2					13	
	Tungsten sealing	74		4	1					15	
	Pharmaceutical	72	1	7	6					11	

**Table 3:** Physical properties of crushed WG.

Property		Refs.
Specific gravity	2.4–2.8 2.51 (Green), 2.52 (Brown)	[40]
Fineness Modulus	4.25 0.44–3.29	[41,42]
Bulk Density	1360 kg/m^3^	[43,44]
Shape Index (%)	30.5	
Flakiness Index	84.3–94.7	[45]

**Table 4:** Mechanical properties of crushed WG.

Property		Refs.
CBR (California bearing ratio) (%)	Approx. 50–75.	[46]
Los Angeles Value (%)	38.4	[43,45]
	24.8–27.8	[44]
	27.7	[47]
Friction Angle	critical = 38 (Loose recycled glass)	[46]
	critical = 51–61 (Dense recycled glass)	

**Table 8:** Summary of the results of past studies on the flexural strength of waste-glass concrete.

Refs.	Type of Composite	Source	Type of Sub.	WG Sub. Ratio%	WG Size (mm)	w/c or w/b	Addit. or Admix.	Flex. str. of Control (MPa)	Outcomes
[74]	SCGC	LCD	F.A	10, 20, & 30 (vol.%)	11.8	0.28	SP	5.1	Changed by +16%, −12%, and −2%, respectively.
[78]	Waste glass concrete	WG	F.A	18, 19, 20, 21, 22, 23, & 24 (vol.%)	0.15–0.6	0.4	SP	4.84	Changed by +5%, +6%, +8%, +7%, +1%, −5% and −6%, respectively.
[79]	Waste glass concrete	CRT	F.A	50 & 100 (vol.%)	≤5	0.35, 0.45, & 0.55	WR & AE	4.4	Decreased by 9%, and 14%, respectively, for w/c of 0.45.
[81]	UHPC	WG	F.A	25, 50, 75, & 100 (wt.%)	≤0.6	0.19	Steel fiber & HRWRA	21	Increased by 2%, 1%, 5%, and 1%, respectively.
[56]	UHPC	CRT	F.A	25, 50, 75, & 100 (vol.%)	0.6–1.18	0.19	Steel fiber, SF, & SP	39	Decreased by 5%, 8%, 18%, and 21%, respectively.
[84]	Waste glass concrete	WG	F.A	5, 15, & 20 (vol.%)	0.15–4.75	0.55	-	4.7	Increased by 6%, 11%, and 15%, respectively.
[55]	SCC	WG	F.A	10, 20, 30, 40, & 50 (vol.%)	0.075–5	0.4	SF & SP	7.4	Decreased by 3%, 11%, 12%, 23%, and 24%, respectively.
[87]	Waste glass concrete	WG	F.A	10, 15, & 20 (vol.%)	0.15–4.75	0.52	-	5.89	Increased by 4%, 7%, and +11%, respectively.
[88]	Waste glass concrete	WG	F.A	15, 20, 30, & 50 (wt.%)	≤5	0.52, 0.57, & 0.67	-	4.5	Decreased by 11%, 22%, 33%, and 44%, respectively, for w/c of 0.57.
[72]	LCDGC	LCD	F.A	20, 40, 60, & 80 (vol.%)	≤4.75	0.38, 0.44, & 0.55	-	3.5	Decreased by 6%, 9%, 10%, and 11%, respectively, for w/c of 0.44.
[126]	Resin concretes	WG	F.A	0–100 (wt.%)	≤2	N.M	Epoxy resin	24.3	Decreased by 1%, for substitution of 100%.

Where: SCGC is self-compacting glass concrete; SCC is self-compacting concrete; UHPC is ultra-high-performance concrete; LCDGC is liquid crystal display glass concrete; LCD is liquid crystal display; CRT is cathode ray tube; WG is waste glass; SP is superplasticizer; HRWRA is a high-range water-reducing agent; WR is water-reducing; AE is air-entraining; SF is silica fume; F.A is fine aggregate; C.A is coarse aggregate; vol. is replacing by volume; wt. is replacing by weight.

## Data Availability

The data used to support the findings of this study are included in the article.
